# Adaptation, Translation and Validation of the Food Allergy Quality of Life Questionnaire-Parent Form (FAQLQ-PF): The Malay Version

**DOI:** 10.3390/children8111050

**Published:** 2021-11-13

**Authors:** Izyana Syazlin Ibrahim, Noorhida Baharudin, Mohamad Rodi Isa, Intan Hakimah Ismail, Mohamed-Syarif Mohamed-Yassin, Intan Kartika Kamarudin, Amir Hamzah Abdul Latiff, Farah Dayana Zahedi, Adli Ali, Azuin Izzati Arshad

**Affiliations:** 1 Department of Primary Care Medicine, Faculty of Medicine, Selayang Campus, Universiti Teknologi MARA, Batu Caves 68100, Selangor, Malaysia; syazlin1306@gmail.com (I.S.I.); syarif8258@uitm.edu.my (M.-S.M.-Y.); 2 Department of Public Health Medicine, Faculty of Medicine, Sungai Buloh Campus, Universiti Teknologi MARA, Sungai Buloh 47000, Selangor, Malaysia; rodi@uitm.edu.my; 3 Department of Paediatrics, Faculty of Medicine & Health Sciences, Universiti Putra Malaysia, Serdang 43400, Selangor, Malaysia; intanhakimah@upm.edu.my; 4 Department of Otorhinolaryngology, Faculty of Medicine, Sungai Buloh Campus, Universiti Teknologi MARA, Sungai Buloh 47000, Selangor, Malaysia; kartika@uitm.edu.my; 5 Allergy and Immunology Centre, Pantai Hospital Kuala Lumpur, Kuala Lumpur 59100, Malaysia; amirlatiff@gmail.com; 6 Department of Otorhinolaryngology, Hospital Canselor Tuanku Muhriz UKM, Kuala Lumpur 56000, Malaysia; farahdayana@ukm.edu.my; 7 Department of Paediatrics, Hospital Canselor Tuanku Muhriz UKM, Kuala Lumpur 56000, Malaysia; adli.ali@ppukm.ukm.edu.my; 8 Department of Otorhinolaryngology, Hospital Sungai Buloh, Sungai Buloh 47000, Selangor, Malaysia; azuin85@gmail.com

**Keywords:** children, food allergy, Malaysia, quality of life, validation

## Abstract

Food allergy has a significant impact on the quality of life (QoL) of children and can be measured using The Food Allergy Quality of Life Questionnaire-Parent Form (FAQLQ-PF). This study aimed to adapt, translate the FAQLQ-PF into Malay and determine the validity and reliability of the translated version. This cross-sectional questionnaire validation study was conducted among parents of children (0 to 12 years old) with food allergies across five sites in Selangor and Kuala Lumpur, Malaysia. The FAQLQ-PF-Malay underwent cross-cultural adaptation, translation, validation (content, face and construct) and reliability assessment. Exploratory factor analysis, internal consistency and test-retest reliability analyses were used to examine its construct validity and reliability. Out of 150 children, the majority were between the age of 7 to 12 years old (41%) and were female (81%). Three subscales were identified, which were: (i) social and dietary implication, (ii) food anxiety and (iii) emotional and physical impact. Four items were eliminated because of weak factor loadings. The Cronbach’s alpha for each subscale ranged from 0.88 to 0.94, with an overall Cronbach’s alpha of 0.95. The intra-class correlation coefficient ranged from 0.54 (95% CI: 0.10–0.77) to 0.97 (95% CI: 0.90–0.99). The 26-item FAQLQ-PF-Malay retained the three-factor structure of the original FAQLQ-PF. The FAQLQ-PF-Malay is a valid and reliable tool to assess the QoL of Malaysian children with food allergies.

## 1. Introduction

Food allergies are a growing problem with an estimated overall worldwide prevalence of 2% to 6% [1,2,3]. The prevalence in children ranges from 6.5% to 8.0% [1,4,5]. Approximately 8% of American children suffer from a food allergy, while 7.1% and 10% of children from Canada and Australia were diagnosed with food allergies, respectively [5,6]. The prevalence in Asia (Taiwan, Singapore and Korea) were comparable to the western countries, ranging from 3.4% to 7.7% [7,8,9]. Among these, 5.3% of food allergies were found in children less than one-year-old [8].

In Malaysia, the most common food allergens among children aged less than two years old were egg white (60%), followed by cow’s milk (44.4%), peanut (36.4%) and soy (25%) [10]. For older children aged two to ten years old, egg white (26.2%) was similarly the most common allergen [10].

Currently, food allergies are managed with strict avoidance of the allergen, with appropriate medications, such as antihistamine and adrenaline injection in the event of unintentional ingestion [11]. However, avoidance is usually impossible, and unintended exposure to allergens may result in anaphylaxis, which can be fatal [12]. The food industry is using precautionary allergen labelling to minimize this risk; however, it is not yet a standard practice and is often ambiguous [13]. All these factors may contribute to poor quality of life among food allergy sufferers.

Food allergies were found to have a significant effect on the QoL of children and their families [14]. It would affect children’s emotional QoL at home and during family activities and social events [14]. Bollinger et al. discovered that food allergies affected meal preparation by the caregiver (60%), family social activities (49%) and school attendance (34%) [15]. Parents of children with food allergies also preferred to home-school their children (10%) [15]. Furthermore, a report revealed significant levels of anxiety and stress among parents [16]. Williams et al. found that these parents experienced distress, guilt, worry and long-standing insecurity concerning their children’s illness [17]. These parents also suffered from extensive anxiety related to potentially life-threatening reactions [18]. In addition, the QoL of children and parents with food allergies was lower compared to other illnesses, such as type 1 diabetes mellitus [19].

Various questionnaires have been discovered to be valid and reliable to measure the QoL of children with food allergies, such as the Paediatric Food Allergy Quality of Life Questionnaire, Food Allergy Independent Measure, Food Allergy Quality of Life Questionnaire-Child Form and the Food Allergy Quality of Life Questionnaire-Parent Form (FAQLQ-PF) [20,21,22]. Among these, the FAQLQ-PF is the only questionnaire that measures the QoL of children aged less than 4 years old. Previous literature reported that food allergies are prevalent in this age group; thus, it is important to measure their QoL [6,8]. The FAQLQ-PF is widely utilized worldwide and has been adapted and translated into nine languages, including Spanish, Turkish, Swedish, Japanese, Portuguese, Dutch, French and Ukrainian [23,24,25,26,27,28,29,30]. It is a valid and reliable tool to assess the QoL of children with food allergies by using parents as a proxy to answer the questionnaire [22]. To the best of our knowledge, FAQLQ-PF has not yet been translated and validated into the Malay language. Thus, this study aimed to adapt, translate and validate the FAQLQ-PF into the Malay language to make it a linguistically and culturally appropriate tool to assess the QoL of children with food allergies in Malaysia.

## 2. Materials and Methods

### 2.1. Study Design and Participants

This cross-sectional questionnaire validation study was conducted among parents of children with food allergies, who completed the questionnaire as a proxy for their children. The participants were recruited from five study sites across the state of Selangor and Kuala Lumpur, Malaysia. These sites were chosen based on the availability of expertise in food allergies and also to ensure adequate representation of participants from various parts of Selangor and Kuala Lumpur. The Paediatric Allergy Clinic, Hospital Pengajar Universiti Putra Malaysia (HPUPM) represented southern Selangor, while the Otolaryngology (ENT) Clinic, Hospital Universiti Teknologi MARA (HUiTM) and Hospital Sungai Buloh represented the Northern part of Selangor. The sites representing Kuala Lumpur were the Paediatric Allergy Clinic, Pantai Hospital Kuala Lumpur (PHKL) and the Allergy Centre, Hospital Canselor Tuanku Muhriz (HCTM).

The inclusion criteria for this study were parents: (i) of children aged 0–12 years old with physician-diagnosed food allergies (clinical manifestation and/or positive skin prick test and/or positive immunoglobulin E (IgE); and (ii) who were able to read and understand the Malay language. Parents of children with chronic medical and psychological/behavioural comorbidities were excluded.

### 2.2. Study Tool

The original English version of the FAQLQ-PF is a 30-item questionnaire with good psychometric properties. The 30 items are framed within three subscales: emotional impact, food anxiety, and social and dietary limitations [22]. There are 13 items in the emotional impact subscale (2, 6, 7, 9, 10, 11, 23, 24, 25, 26, 27, 28, 30), eight items in the food anxiety subscale (1, 4, 5, 16, 17, 20, 21, 29) and nine items in the social and dietary limitation subscale (3, 8, 12, 13, 14, 15, 18, 19, 22). The reliability coefficients (Cronbach alpha) of all subscales are 0.94, 0.92 and 0.91, respectively [22]. The overall Cronbach’s alpha is 0.91 [22]. The intraclass correlation coefficient (ICC) values exceed 0.70 for all the items, indicating good reproducibility [22]. The FAQLQ-PF also demonstrated concurrent validity with a significant correlation with the Child Health Questionnaire (CHQ-28), another questionnaire that measures children’s quality of life (r = 0.69–0.77, *p* < 0.01) [22].

The questionnaire is divided into three sections according to age, where older children have more questions compared to the younger ones. The age was grouped into three categories, which were the infant and toddlers (<4 years old), pre-school age (4 to 6 years old) and primary-school age (7–12 years old). This questionnaire was designed to accommodate various developmental and social circumstances of children from varying ages as they may encounter different challenges during each milestone. There are 14 items for children aged less than four years. In addition to the 14 items, children aged four to six years have an extra 12 items (total of 26 items), while children aged seven to 12 have an extra 16 items (total of 30 items) [22]. All items are scored on a 7-point Likert scale from 0 (not at all troubled) to 6 (extremely troubled) [22]. The total scores are divided by the number of items answered, giving a range of scores from 0 to 6, with higher values indicating a poorer quality of life [22]. The author of the original English version of FAQLQ-PF had granted permission for it to be adapted and translated into the Malay language.

In addition to the FAQLQ-PF, demographic and clinical characteristics of the participants were also collected. The severity of food allergies was defined based on the Australasian Society of Clinical Immunology and Allergy guideline [31]. Mild to moderate allergic reactions were defined based on at least one of the following signs or symptoms: swelling of lips, face, eyes, itching in the mouth, hives or welts, abdominal pain or vomiting [31]. In severe allergic reactions, the signs or symptoms are recognizable by at least one of the following manifestations: difficulty breathing, swelling of the tongue, difficulty swallowing, wheeze or persistent cough, difficulty talking and/or hoarseness, persistent dizziness or loss of consciousness or pale and floppy for young children [31].

### 2.3. Conduct of the Study

This study was divided in two parts: Part 1 was the cross-cultural adaptation, translation and face validation; Part 2 was the field testing and psychometric analysis.

#### 2.3.1. Part 1: Cross-Cultural Adaptation, Translation and Face Validation

The content validation was performed by a panel of seven experts. This panel consisted of two paediatric allergy specialists, three family medicine specialists, one paediatrician and one child psychologist. The content validation was conducted via electronic mail. Explicit instructions on how to conduct the tasks were given to each expert. They were requested to comment on the questionnaire based on the relevance and the clarity of each item in each domain.

The content validity index (CVI) was used to determine the content validity of the FAQLQ-PF [32]. CVI assesses the relevance of each item to the domain [32]. The experts were requested to rate each item on a scale from 1 to 4 (1 = not relevant, 2 = somewhat relevant, 3 = quite relevant, 4 = highly relevant). Two forms of CVI were used in this study. These were CVI for the items (I-CVI) and CVI for the scale (S-CVI) [32]. The I-CVI was determined by the proportion of content experts giving the item a relevance rating of 3 or 4 (the number of experts rating 3 or 4 divided by the total number of experts) [32]. S-CVI was calculated by dividing the total of the I-CVI scores by the number of items (total of I-CVI/number of items) [32]. I-CVI and S-CVI values >0.8 indicate that the item is relevant to the domain [32,33,34]. The panel was also asked regarding the clarity of each item, and their suggestions to improve the items. Their comments were documented by the lead researcher.

The English version of FAQLQ-PF was translated into the Malay language according to the recommendations from Beaton et al. [35]. The forward translation into Malay was conducted by two independent bilingual individuals (one linguistic expert from the Academy of Language Studies, UiTM, and one family medicine specialist). The FAQLQ-PF Malay-translated version (FAQLQ-PF M-t) was produced following a detailed discussion between both translators and the research team in the process of reconciliation. The reconciled version was back-translated by two independent bilingual individuals (one paediatrician and one linguistic expert from the UiTM Academy of Language Studies). The back translators were not aware of the original, English version of FAQLQ-PF. The translated versions were discussed by the research team (medical professionals and language experts) to produce the final harmonized preliminary Malay version (FAQLQ-PF-HM) [35].

**Figure 1:**
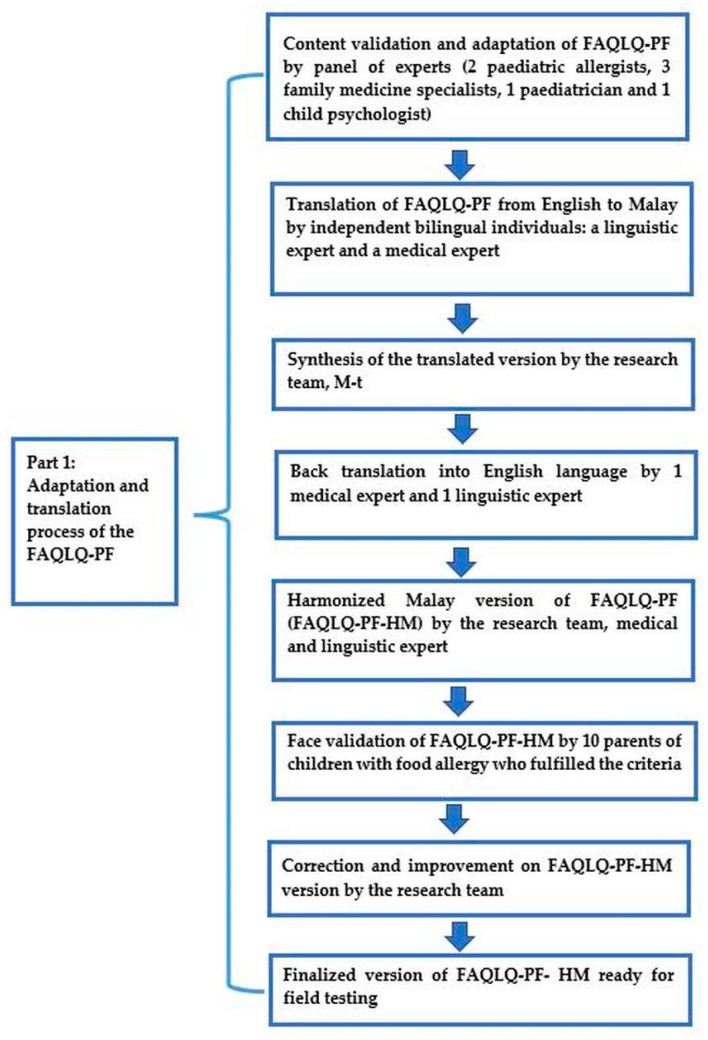
Part 1 flow chart. FAQLQ-PF-HM: Food Allergy Quality of Life-Parent Form-Harmonized Malay.

The FAQLQ-PF-HM version was pre-tested on 10 parents of children with food allergies who were eligible according to the inclusion and exclusion criteria [36]. The questionnaire was self-administered. They were asked to comment on the wording, clarity and overall structure of the questionnaire. The time needed to complete the questionnaire was also documented. The research team corrected and fine-tuned the FAQLQ-PF-HM based on the outcome from the face validity testing to ensure that items were clear and comprehensible. Fine-tuning of the FAQLQ-PF-HM by the research team was conducted. This refined FAQLQ-PF-HM was ready to undergo field testing. These 10 participants were not invited for Part 2 of this study. The steps carried out in Part 1 of the study are outlined in Figure 1.

#### 2.3.2. Part 2: Field Testing and Psychometric Analysis

The sample size was calculated using an item-to-subject ratio. Pallant recommended a minimum sample size of 150 or at least a 1:5 ratio [37]. FAQLQ-PF contains 30 items; therefore, the minimum number of recruitments was 150. After considering a 20% non-responder rate, the study aimed to recruit 180 participants.

This study utilized the convenience sampling method. This method was chosen because of the time and resource constraints to conduct the study. To minimize sampling bias, each consecutive patient was approached to participate on data collection days.

Participants were recruited over the duration of five months between December 2020 to April 2021 at all five study sites. Clinic appointments, skin prick tests appointments and IgE lab results were used to identify patients with food allergies from each study site. Their eligibility (clinical manifestation and/or positive skin prick test and/or positive IgE test) was confirmed through a review of their medical records.

Eligible parents were invited to participate face-to-face in the clinic’s waiting room. Parents who were interested were provided with the study information sheet containing pertinent information, such as the background and purpose of the study, study procedure and confidentiality status. Those who were eligible and agreed to take part were recruited. Written informed consent was obtained. The participants were asked to self-administer the FAQLQ-PF-HM. Once completed, the questionnaires were checked for completeness.

In view of the worsening Coronavirus Disease 2019 (COVID-19) pandemic in Malaysia, where clinic visits were reduced significantly, the recruitment method was subsequently revised to phone interviews. The invitations to participate in phone interviews were sent via short message service (SMS). Similar to face-to-face recruitment, the participants were given the study information sheet. Those who were interested in participating were given an appointment for phone interviews. The researcher-administered method was used for data collection. Those who were eligible and consented were recruited. Verbal informed consent was obtained.

Thirty participants from those who were previously recruited were given the same questionnaire to complete or had another phone interview using the same questionnaire after two weeks [38].

### 2.4. Statistical Analysis

Data were analysed using the latest version of IBM Statistical Package for the Social Sciences (SPSS) version 27 (IBM Corp., Armonk, NY, USA). For descriptive analysis, data were presented as the mean or median based on normality testing. Categorical variables were described in numbers and percentages. The construct validity was assessed using exploratory factor analysis (EFA).

The suitability of data for factor analysis was assessed based on Kaiser–Meyer–Olkin (KMO) values of >0.6, and a significant Bartlett’s Test of Sphericity with the *p*-value < 0.05. A significant Bartlett’s Test of Sphericity indicated the presence of correlations between the items, while KMO values >0.6 indicated that the sample was adequate for each variable to assess the proportion of variance in the variables that might be caused by the underlying factors [39].

For EFA, the principal axis factoring (PAF) extraction method was chosen as an assumption that multivariate normality was violated [40]. The oblique rotation (promax) method was used as there were correlations between the factors [39].

The Kaiser’s criterion, scree plot and parallel analysis using Monte Carlo PCA were performed to determine the number of factors to be retained. For Kaiser’s criterion, an eigenvalue greater than 1 determined the number of factors that should be extracted [39]. According to the scree plot, the data above the point of inflexion determined the number of factors to be retained [39]. The parallel analysis with the Monte Carlo simulation method identified factors by randomly generating the data that matched the sample size and the total of items in the questionnaire [41]. The factors were retained if the eigenvalues from parallel analysis were greater than the eigenvalues generated from factor analysis [42].

An acceptable factor loading is based on the number of participants [39]. Field recommended a factor loading of more than 0.512 for a sample size of 100, and a factor loading of more than 0.364 for a sample size of 200 [39]. This study recruited a total of 150 participants; thus, items with a factor loading of 0.40 or greater were considered appropriate for this study. The final factor structure was based on three criteria, which were items with factor loading of ≥0.4, no or minimal cross-loading (items that load 0.32 or higher on two factors) and all factors with a minimum of three items [40].

The internal consistency was analysed using Cronbach’s alpha coefficient and corrected item-total correlations. A value >0.70 for Cronbach’s alpha coefficient was considered reliable [39]. The corrected item-total correlations are the correlations between individual items to the sum scale of the questionnaire. Correlations of *r* ≥ 0.10 was considered weak, *r* ≥ 0.30 moderate and *r* ≥ 0.50 strong [39]. Test-retest reliability was assessed using ICC [43]. A value ≥ 0.7 indicated a good test-retest reliability [44].

**Figure 2:**
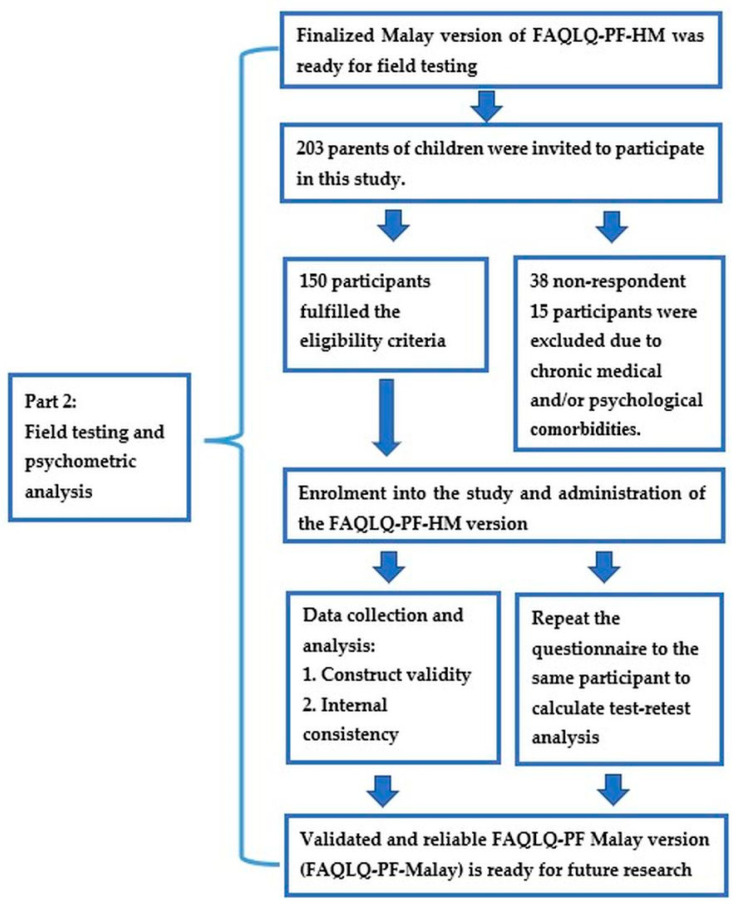
Part 2 study conduct flow chart. FAQLQ-PF-HM: Food Allergy Quality of Life-Parent Form-Harmonized Malay.

The conduct for Part 2 of this study is outlined in Figure 2.

## 3. Results

### 3.1. Participant Demographics

In total, 203 parents of children were invited to take part in this study. For face-to-face recruitment, 41 participants were approached, and 41 participants (100%) were recruited. A total of 162 SMS messages were sent to potential participants, and 124 (77%) responded and agreed for phone interviews. Following phone interviews, 15 children were subsequently excluded due to chronic medical and/or psychological comorbidities. Thus, 109 children were recruited via phone interviews. In total, 150 participants were eligible and agreed to participate in this study (72 from paediatric allergy clinic HPUPM, 17 from paediatric allergy clinic PHKL, 36 from ENT Clinic HUiTM Sungai Buloh, 10 children from ENT clinic Hospital Sungai Buloh and 15 from Allergy Centre, HCTM). The median age (IQR) was 5 (7) years with the majority of children being female (80.7%). The participant’s demographics and clinical characteristics are shown in Table 1.

### 3.2. Cross-Cultural Adaptation, Translation, and Face Validation

Seven experts contributed to the content validation process. I-CVI was found to be >0.80 for all items except for items 5 and 10. For item 5 “Concerned that I am worried that he/she will have a reaction to food”, three of the experts believed that this item was not developmentally appropriate for children younger than four years old. Following deliberation, the experts agreed that this item was developmentally appropriate for children aged four and above. Thus, this item was moved to the section for children aged four to 12 years old. As for item 10 “Having to grow up more quickly than other children of his/her age”, three of the experts agreed that this question sounded ambiguous. The panel felt that the participants may interpret this item literally, i.e., the children may physically grow faster than other children his/her age. Therefore, the expert panel decided to rephrase the item to “Having to become emotionally more mature than other children of his/her age”. After modifications, the items’ relevancy, clarity and comprehensiveness for each domain were assessed. The final I-CVI scores for each item were ≥0.86, with a S-CVI score of 0.99.

The questionnaire underwent forward and backward translations. In each process, the research team, expert panel and translators were consulted, and a consensus on the translated FAQLQ-Malay-harmonized version was reached.

For face validation, all ten participants deemed that the FAQLQ-Malay-harmonized version was simple and easy to read. The time taken to answer the questionnaire was 10 min.

### 3.3. Psychometric Analysis

The KMO value was 0.84, and a significant Bartlett’s Test of Sphericity (*p* < 0.001) indicated that the data were suitable for factor analysis. PAF with promax rotation was used for extraction in the EFA as there were correlations between the factors. Most of the factor correlation matrix were above 0.30.

Initially, a total of five factors that explained 65.9% of the variance showed eigenvalues above 1.0. The communalities values were above the minimum cut-off point value of 0.40. Further analysis using the scree plot showed an inflexion at factor five, suggesting four factors to be retained. The parallel analysis with Monte Carlo PCA proposed three factors to be retained. The three- to five-factor solutions were explored repeatedly. The three-factor solution was considered to be the most conceptually appropriate for the FAQLQ-PF Malay version.

The data were reanalysed by setting the number of factors at three, and a minimum factor loading of 0.40. The total variance of the three-factor solution was 58.5%. Four items (10, 23, 27, 30) did not meet the minimum criteria of having a factor loading of ≥0.40. Thus, the items were removed in a stepwise manner, starting with the item with the lowest factor loading (item 23), followed by the removal of item 10, 27 and 30. Following the removal of these four items (10, 23, 27, 30), the KMO value obtained was 0.85, which showed a slight improvement from the initial 30-item factor solution (KMO = 0.84). The Bartlett’s Test of Sphericity was significant with *p* < 0.001. The three factors explained 60.4% of the total variance.

Four items cross-loaded onto factor 1 and factor 3. These were items 18, 19, 21 and 25. Items 18, 19 and 25 were retained on their priori factor. Item 21 was considered to fit conceptually with factor 3 and was allocated to this factor.

The final factor solution consisted of 26 items, framed within three constructs. This factor solution was deemed to be the most interpretable, conceptionally appropriate and fulfilled the requirements of factor loading ≥0.4, minimal cross-loading and all factors had a minimum of three items [40].

The final three-factor solution is shown in Table 2. Factor 1 was labelled as “social and dietary implication” and consisted of 11 items (5, 9, 11, 12, 13, 14, 15, 16, 18, 19, 22). Factor 2 consisted of eight items (1, 4, 17, 20, 24, 26, 28, 29) and was labelled as “food anxiety”. Factor 3 consisted of seven items (2, 3, 6, 7, 8, 21, 25) and was named “emotional and physical impact”. This final three-factor solution explained 60.4% of the total variance.

### 3.4. Reliability

In terms of reliability, the overall Cronbach’s alpha coefficient of the FAQLQ-PF-Malay was 0.95, which indicated excellent internal consistency. The corrected item-total correlation coefficients ranged from 0.50 to 0.79, which showed a moderate to strong correlation of all 26 items. The Cronbach’s alpha for subscale 1 (social and dietary limitation), 2 (food anxiety) and 3 (emotional and physical impact) were 0.94, 0.88 and 0.91, respectively. As for test-retest reliability, the ICC values ranged from 0.54 (95% CI: 0.10–0.77) to 0.97 (95% CI: 0.90–0.99), which indicated high reproducibility. Table 3 shows Cronbach’s alpha values for the three subscales and the intraclass correlation coefficients (ICC) for each item.

## 4. Discussion

The FAQLQ-PF is widely utilized worldwide and has been shown to have good psychometric properties [22,23,25]. To our knowledge, this study was the first to cross-culturally adapt, translate and validate the FAQLQ-PF into the Malay language. This study has demonstrated that the FAQLQ-PF-Malay is valid and reliable as a tool to assess the quality of life of Malaysian children with food allergies, using their parents as a proxy. Three subscales were identified from the EFA. The subscales were: (i) social and dietary implication, (ii) food anxiety and (iii) emotional and physical impact. The final version of the FAQLQ-PF-Malay consisted of 26 items with good construct validity and reliability.

In the content validation process, item 5 “Concerned that I am worried that he/she will have a reaction to food”, which was previously in the section for children 0 to 4 years old, was moved to the sections for children aged four years and older. The panel felt that children younger than four are egocentric and cognitively incapable of understanding other people’s perspectives [45]. From the age of four years old, the children would start to understand other people’s feelings [45]. Thus, the panel agreed that item 5 would fit more appropriately in the older-aged groups. Following correction, the I-CVI score for this item was 1.

As for item 10 “Having to grow up more quickly than other children of his/her age”, the experts panel believed that this sentence is too ambiguous. A direct translation into Malay would mean that the children would “physically grow up more quickly than other children”. The panel interpreted this item as children would have to “grow up” psychologically and emotionally; hence, this item was rephrased to “Having to become emotionally more mature than other children of his/her age”. The I-CVI prior to revision was 0.57. Following discussion and reconciliation, the final I-CVI score for this item improved to 1.

The FAQLQ-PF-Malay consisted of three subscales (social and dietary implication, food anxiety, emotional and physical impact) with a total explained variance of 60.4%. This is similar to the original FAQLQ-PF (social and dietary limitations, food anxiety, emotional impact) and other validation studies from Japan, Turkey and Ukraine [22,23,25,30].

Four items (10, 23, 27, 30) were eliminated because of their weak factor loadings (<0.40) on their respective scales. Ten items (3, 5, 8, 9, 11, 16, 21, 24, 26, 28) loaded on a different factor compared to their priori factor. These changes probably occurred due to the influence of translation and the differences in perception in different cultures. Although the characteristics of the items are similar, respondents’ perceptions may vary between cultures and countries [46]. The emotional impact domain of FAQLQ-PF-Malay consisted of seven items, compared to the original FAQLQ-PF, which has 13 items. The reduced number of items in this domain did not have a significant impact on the questionnaire because the items were distributed in all age groups. Furthermore, this 7-item domain demonstrated excellent reliability with Cronbach alpha value of 0.91.

The final FAQLQ-PF-Malay consisted of 26 items compared to the original FAQLQ-PF that had 30 items [22]. The findings of the 26-item FAQLQ-PF-Malay have not been replicated previously as other translation and validation studies used different methods for validity testing (concurrent and discriminant validity) for their version of the FAQLQ-PF [23,24,25,27,30]. These studies retained all 30 items, and the concurrent validity of the translated FAQLQ-PF was determined through correlations with other quality of life questionnaires, such as the CHQ-28 and the Food Allergy Independent Measure [23,24,25,27,30]. Nevertheless, the FAQLQ-PF-Malay is more efficient as it has less items while still being able to maintain its validity and reliability. It would take less time for participants to complete the questionnaire and would reduce the risk of missing data.

The final FAQLQ-PF-Malay demonstrated excellent internal consistency with an overall Cronbach’s alpha of 0.95 and for each subscale (0.94, 0.88 and 0.91). This finding is consistent with other studies that found Cronbach’s alpha values ranging from 0.72 to 0.97 [22,23,24,25,27,30]. The ICC values for the FAQLQ-PF-Malay ranged from 0.54 (95% CI: 0.10–0.77) to 0.97 (95% CI: 0.90–0.99). This indicates that the FAQLQ-PF-Malay had high stability. Other studies also demonstrated similar findings, with an ICC of >0.6 [22,23,24,25]. The consistent findings from all these studies demonstrated that the translated versions of the FAQLQ-PF are reliable for use in those cultures and languages.

### Strengths, Limitations and Implication for Future Research

Our study was the first to cross-culturally adapt, translate and validate the FAQLQ-PF into the Malay language following established guidelines, such as COnsensus-based Standards for the selection of health Measurement INstruments (COSMIN) checklist [47]. This study recruited participants from five study sites across various parts of Selangor and Kuala Lumpur, which provided a good representation of Malaysian children with food allergies from these two states in Malaysia. The study population was also well distributed, thus providing good representation of children from all age groups. There were more females (81%) included in this study. To date, the national prevalence of food allergies in Malaysia, including prevalence according to gender, is still lacking. Another small study in Kuala Lumpur [10] did not report on the gender distribution of their study participants; thus, our findings could not be compared to existing literature. This finding may reflect the true prevalence of food allergies among our study population; however, this may also occur due to sampling bias.

In terms of the study limitation, selection bias may occur where participants may not adequately represent other children with food allergies in Malaysia. To overcome this, future studies should include participants from various clinics from each state in Malaysia to ensure adequate representation and generalisability of this questionnaire for other children with food allergies in Malaysia. In addition, this questionnaire should also be translated into other common languages in Malaysia, i.e., Mandarin and Tamil, to cater to the needs of other major ethnic communities in Malaysia.

Initially, the data collection method for this study was via self-administered questionnaires. However, the COVID-19 pandemic led to difficulties in recruiting the required sample size. The face-to-face self-administered questionnaire method was not feasible due to the time constraint to complete this study. Hence, data collection was changed to phone interviews, which may introduce response bias. The participants recruited from phone interviews may answer the questionnaire according to what they perceived to be favourable, which could have affected their QoL score. The QoL score from these participants, however, could also be influenced by other factors, such as the severity of food allergies and the number of allergens. The association between these factors and the QoL of the children is beyond the scope of this article and may be explored in future research.

Although the sample-to-variable ratio of 1:5 was adequate for EFA in this study, a bigger sample size with a minimum of 300 is needed for Confirmatory Factor Analysis (CFA) and Rasch Model Analysis [48]. The CFA and Rasch Model Analysis would further confirm the factor structure of FAQLQ-PF-Malay; thus, future studies should recruit at least 300 participants to enable these analyses [48].

The FAQLQ-PF-Malay may be used for future research to assess the quality of life of children with food allergies in Malaysia. The factors associated with the QoL of these children could also be studied, which would provide insights for various stakeholders, such as parents of children with food allergies, healthcare professionals and educators in childcare and schools. Intervention strategies, such as health promotion to improve awareness regarding food allergies, can then be developed to improve the QoL of these children. Furthermore, the FAQLQ-PF-Malay may also be used as one of the tools to measure the effectiveness of these interventions in improving the quality of life of Malaysian children with food allergies.

## 5. Conclusions

The FAQLQ-PF-Malay is a valid and reliable tool to assess the QoL of Malaysian children with food allergies. To improve the generalizability of this tool, future research should include participants from various clinics from each state in Malaysia. To confirm the construct of the FAQLQ-PF-Malay, further evaluation with CFA and Rasch Model analysis should be performed.

## Figures and Tables

**Table 1:** Demographic and clinical characteristics of children with food allergies (*n* = 150).

Variables	*n* (%)	Median (IQR)
**Age (years), median (IQR)**		5 (7)
**Age Group (years)**		
<4 years old 4–6 years old 7–12 years old	57 (38%) 32 (21%) 61 (41%)	
**Gender**		
Male Female	29 (19%) 121 (81%)	
**Ethnicity**		
Malay Chinese Indian	141 (94%) 7 (4.7%) 2 (1.3%)	
**Severity of food allergy**		
Mild to moderate Severe	95 (63%) 55 (37%)	
**Number of food Allergens**		
1 2 3 4 ≥5	38 (25%) 41 (27%) 21 (14%) 21 (14%) 29 (20%)	
**Type of food allergens**		
Cow’s Milk/Dairy Egg Soy Wheat Peanut Tree nut Fish Shellfish Others	60 (40%) 68 (45%) 26 (17%) 24 (16%) 34 (23%) 16 (11%) 29 (19%) 70 (47%) 27 (43%)	

**Table 2:** Rotated Pattern Matrix on the final three-factor solution.

Item	Factor 1	Factor 2	Factor 3
Item 14: “Because of food allergy, my child’s ability to take part has been limited in social activities in other people’s houses (sleepovers, parties, playtime)”/ “*Disebabkan alahan makanan, keupayaan anak saya untuk mengambil bahagian telah terbatas dalam aktiviti sosial di rumah orang lain. (bermalam, majlis keraian, masa bermain*”	0.903		
Item 15: “Because of food allergy, my child’s ability to take part has been limited in preschool/school events involving food (class parties/treats/ lunchtime)”/“*Disebabkan alahan makanan, keupayaan anak saya untuk mengambil bahagian telah terbatas dalam acara prasekolah/sekolah yang melibatkan makanan* *(majlis keraian kelas/makanan ringan/waktu makan tengah hari*”	0.737		
Item 13: “Because of food allergy, my child’s social environment is restricted because of limitations on holiday destinations we can safely go to as a family”/ “*Disebabkan alahan makanan, persekitaran sosial anak saya terhad disebabkan batasan terhadap destinasi percutian yang kami sekeluarga boleh pergi dengan selamat*”	0.731		
Item 12: “Because of food allergy, my child’s social environment is restricted because of limitations on restaurants we can safely go to as family”/“*Disebabkan alahan makanan, persekitaran sosial anak saya terhad disebabkan batasan terhadap restoran yang kami sekeluarga boleh pergi dengan selamat*”	0.688		
Item 22: “Because of food allergy, my child feels frustrated by social restrictions”/“*Disebabkan alahan makanan, anak saya berasa kecewa dengan had sosial*”	0.660		
Item 19: “Because of food allergy, my child feels upset that family social outings have been restricted by the need to plan ahead”/“*Disebabkan alahan makanan, anak saya berasa sedih kerana acara sosial keluarga telah terbatas oleh keperluan untuk merancang lebih awal*”	**0.636**		0.443
Item 9: “Because of food allergy, my child has been negatively affected by receiving more attention than other children of his/her age”/“*Disebabkan alahan makanan, anak saya telah terkesan secara negatif dengan mendapat perhatian yang lebih berbanding kanak-kanak lain yang seusia dengannya*”	0.605		
Item 5: “Because of food allergy, my child feels concerned that I am worried that he/she will have a reaction to food”/“*Disebabkan alahan makanan, anak saya berasa khuatir bahawa saya bimbang dia akan mengalami reaksi terhadap makanan*”	0.545		
Item 16: “Because of food allergy, my child feels worried when going to unfamiliar places”/“*Disebabkan alahan makanan, anak saya berasa bimbang apabila pergi ke tempat yang dia tidak biasa*”	0.542		
Item 11: “Because of food allergy, my child has been negatively affected by his/her environment being more restricted than other children of his/her age”/ “*Disebabkan alahan makanan, anak saya telah terkesan secara negatif dengan persekitarannya yang lebih terbatas daripada kanak-kanak lain yang seusia dengannya*”	0.520		
Item 18: “Because of food allergy, my child feels ‘left out’ in activities involving food”/“*Disebabkan alahan makanan, anak saya berasa ‘terpinggir’ dalam aktiviti yang melibatkan makanan.*”	**0.497**		0.456
Item 17: “Because of food allergy, my child feels concerned that he/she must always be cautious about food”/“*Disebabkan alahan makanan, anak saya berasa khuatir bahawa dia mesti sentiasa berwaspada tentang makanan*”		0.745	
Item 24: “Because of food allergy, my child is more cautious in general than other children of his/her age”/“*Disebabkan alahan makanan, anak saya secara amnya lebih berhati-hati berbanding kanak-kanak lain yang seusia dengannya*”		0.706	
Item 28: “Because of food allergy, my child feels many people do not understand the serious nature of food allergy”/“*Disebabkan alahan makanan, anak saya berasa ramai orang tidak memahami tahap keseriusan alahan makanan*”		0.703	
Item 1: “Because of food allergy, my child feels worried about food”/“*Disebabkan alahan makanan, anak saya berasa bimbang tentang makanan*”		0.699	
Item 20: “Because of food allergy, my child feels concerned about accidentally eating an ingredient to which he/she is allergic”/“*Disebabkan alahan makanan, anak saya berasa khuatir jika tidak sengaja termakan bahan makanan yang dia alah*”		0.584	
Item 29: “Because of food allergy, my child feels concerned by poor labelling on food products”/“*Disebabkan alahan makanan, anak saya berasa khuatir dengan pelabelan yang tidak lengkap pada bahan makanan*”		0.514	
Item 4: “Because of food allergy, my child feels afraid to try unfamiliar foods”/ “*Disebabkan alahan makanan, anak saya berasa takut mencuba makanan yang dia tidak biasa*”		0.509	
Item 26: “Because of food allergy, my child wishes his/her food allergy would go away”/“*Disebabkan alahan makanan, anak saya berharap agar alahan makanannya akan hilang*”		0.454	
Item 7: “Because of food allergy, my child experiences emotional distress”/ “*Disebabkan alahan makanan, anak saya mengalami tekanan emosi*”			0.811
Item 6: “Because of food allergy, my child experiences physical distress”/ “*Disebabkan alahan makanan, anak saya mengalami tekanan fizikal*”			0.802
Item 3: “Because of food allergy, my child feels frustrated by dietary restriction”/“*Disebabkan alahan makanan, anak saya berasa kecewa dengan pemakanan yang terbatas*”			0.771
Item 2: “Because of food allergy, my child feels different from other children”/ “*Disebabkan alahan makanan, anak saya berasa berbeza daripada kanak-kanak lain*”			0.745
Item 8: “Because of food allergy, my child has a lack of variety in his /her diet”/ “*Disebabkan alahan makanan, anak saya mempunyai kekurangan variasi dalam pemakanannya*”			0.711
Item 25: “Because of food allergy, my child is not as confident as other children of his/her age in social situations”/“*Disebabkan alahan makanan, anak saya kurang berkeyakinan dalam situasi sosial berbanding kanak-kanak lain yang seusia dengannya*”	0.639		**0.457**
Item 21: “Because of food allergy, my child feels worried when eating with unfamiliar adults/children”/“*Disebabkan alahan makanan, anak saya berasa Bimbang ketika makan dengan orang dewasa/kanak-kanak yang dia tidak biasa*”	0.452		**0.417**

Principal axis factoring, promax rotation; Fixed number of factors = 3; Suppressed small coefficients, absolute value below 0.4; Bold: The factor which cross-loaded items (item 18,19,21,25) are allocated to

**Table 3:** Cronbach’s alpha values for each subscale and intraclass correlation coefficients (ICCs) for each item.

Subscale	Number of Items	Cronbach’s Alpha	Corrected Item-Total Correlation	Cronbach’s Alpha If Item Deleted	Intraclass Correlation Coefficient, ICC (95%CI)
**Social and dietary implication**	11	0.94			
Item 5			0.74	0.94	0.84 (0.53–0.94)
Item 9			0.63	0.94	0.86 (0.72–0.93)
Item 11			0.70	0.94	0.81 (0.64–0.90)
Item 12			0.81	0.93	0.85 (0.71–0.92)
Item 13			0.79	0.94	0.87 (0.74–0.93)
Item 14			0.80	0.93	0.93 (0.87–0.97)
Item 15			0.80	0.94	0.90 (0.71–0.96)
Item 16			0.70	0.94	0.85 (0.56–0.95)
Item 18			0.80	0.93	0.90 (0.74–0.97)
Item 19			0.80	0.94	0.90 (0.71–0.96)
Item 22			0.69	0.94	0.89 (0.54–0.97)
**Food anxiety**	8	0.88			
Item 1			0.58	0.88	0.79 (0.60–0.89)
Item 4			0.60	0.87	0.77 (0.56–0.88)
Item 17			0.68	0.87	0.85 (0.55–0.95)
Item 20			0.70	0.86	0.81 (0.46–0.94)
Item 24			0.77	0.86	0.88 (0.64–0.96)
Item 26			0.55	0.88	0.97 (0.90–0.99)
Item 28			0.75	0.86	0.93 (0.72–0.98)
Item 29			0.63	0.87	0.89 (0.60–0.97)
**Emotional and physical impact**	7	0.91			
Item 2			0.77	0.90	0.80 (0.60–0.90)
Item 3			0.71	0.89	0.54 (0.10–0.77)
Item 6			0.79	0.90	0.78 (0.58–0.90)
Item 7			0.84	0.88	0.79 (0.60–0.90)
Item 8			0.66	0.90	0.83 (0.68–0.91)
Item 21			0.69	0.90	0.59 (0.25–0.86)
Item 25			0.66	0.90	0.74 (0.28–0.91)
**Overall**	26	0.95			

## Data Availability

Data are stored at the Department of Primary Care Medicine, Universiti Teknologi MARA, Selangor, Malaysia. Data will be supplied only upon reasonable request and will be protected by data protection regulations.
